# Limiting support for environmental policies: Unfairness is a more critical barrier than cost and ineffectiveness

**DOI:** 10.1007/s13280-024-02074-9

**Published:** 2024-09-17

**Authors:** Magnus Bergquist

**Affiliations:** Department of Psychology, University of Gothenburg, Haraldsgatan 1, 413 14 Göteborg, Sweden

**Keywords:** Carbon tax, Environmental policy, Experiment, Fairness, Meat tax, Support

## Abstract

Costs and policy-specific beliefs, such as effectiveness and fairness, are central factors for supporting environmental taxes. Less is known about how much each of these factors is limiting support. Across four experiments, I investigate to which extent high costs, ineffectiveness, and unfairness constrain support for environmental taxes. Results consistently demonstrate that perceived unfairness poses a greater barrier to support than extensive costs or ineffectiveness. These findings were robust across three environmental taxes (meat tax, plastic tax, and carbon tax), across three cultures (USA, UK, and India), and were replicated using a representative US sample. Furthermore, delving deeper into the consequences of perceived unfairness, results showed that distributional unfairness was a stronger barrier to support than procedural unfairness. Beyond limiting support, being presented with an unfair tax proposal led participants to perceive the tax as less effective and to express lower trust in policymakers than when receiving a fairer tax proposal.

**Supplementary Information:**

The online version contains supplementary material available at 10.1007/s13280-024-02074-9.

## Introduction

Policy instruments such as taxes and regulations can potentially promote climate change mitigation by reducing CO_2_ emissions (Köppl and Schratzenstaller 2023; Sterner 2012) or preventing environmental damage caused by plastics and plastic littering (Groh et al. 2019; Shen et al. 2020; UNEP 2023). However, implementing environmental taxes carries the risk of public resistance (Driscoll 2023; Povitkina et al. 2021). How, then, should environmental policies be designed to gain support? Meta-analytic evidence suggests that policy-specific beliefs about fairness and effectiveness are the most important determinants for supporting climate change taxes and laws (Bergquist et al. 2022). Implying that environmental taxes perceived as fair or effective in reducing CO_2_ emissions are strongly positively related to support (e.g., Dreyer and Walker 2013; Savin et al. 2020; see also Zimm et al. 2024). In this context, perceived fairness refers to the extent to which a policy instrument is considered fair, both in terms of distribution and procedure (Maestre-Andrés et al. 2019; Jagers et al. 2010). For example, a carbon tax might be perceived as distributionally unfair to people who rely on driving, and a congestion charge might be seen as procedurally unfair if implemented in an “undemocratic” manner (Nilsson et al. 2016; Povitkina et al. 2021). Perceived effectiveness refers to the belief that a policy can successfully achieve its specific goal, such as reducing carbon emissions (Ejelöv and Nilsson 2020). Additionally, in the context of taxation, the cost of the tax has also been shown to be strongly linked to public support (e.g., Coleman et al. 2023; Keizer et al. 2023).

Perceived fairness is central to several environmental policies, including those for low-carbon passenger transportation (Thaller et al. 2023), personal carbon trading (Pitkänen et al. 2022), and on-site wastewater treatment and reuse (Kollmann et al. 2024). However, fairness is a multi-faceted concept. Describing a proposed policy as “unfair” could refer to distributional, procedural, or personal concerns. This might involve the unequal distribution of costs, non-transparent handling of taxes, or restrictions of personal needs (e.g., Maestre-Andrés et al. 2019; Povitkina et al. 2021). When proposing an environmental tax, research offers straightforward advice for gaining support, such as implementing re-distribution schemes (e.g., Carattini et al. 2018), increasing transparency (Povitkina et al. 2021), and proposing subsidies rather than taxes (Coleman et al. 2023).

Although these insights are valuable, much of the research on the determinants for support for environmental policies is limited by using cross-sectional designs (Kallbekken 2023). The current work aims to advance past research by conducting a series of experimental studies using between-subjects designs. In these studies, participants are randomly assigned to different experimental conditions that manipulate three key determinants: the costs, ineffectiveness, and unfairness of policy proposals.

The question of causality is indeed important for the field of policy acceptance. Although past research has shown that support for policies is consistently and strongly related to perceived ineffectiveness and perceived unfairness (e.g., Bergquist et al. 2022; Ejelöv and Nilsson 2020), cross-sectional studies cannot establish that these determinants causality influence support. It is possible that support for, for example, a carbon tax leads people to rationalize their perceptions of fairness to align with their support. Both classical and contemporary psychological research suggests that people tend to adjust their attitudes to be consistent with their overt behavior (e.g., Festinger 1957; Regan and Kilduff 1988; Jarcho et al. 2011). In a series of Experiments, I examine how induced cost, ineffectiveness, and unfairness relate to support for environmental taxes.

Past research has provided valuable insight by assessing various policies such as carbon tax, renewable energy, and road pricing (e.g., Ballew et al. 2019; Bamberg and Rölle 2003, Jagers et al. 2018). However, most studies tend to focus on a single type of policy tool (see Bergquist et al. 2022 for an overview). In this paper, I examine three environmental taxes: a carbon tax, a tax on plastics, and a tax on meat. Carbon taxation is widely regarded as one of the most cost-effective methods for reducing greenhouse gas emissions and is frequently recommended by environmental economists and policy researchers (e.g., Sterner and Coria 2013; Stiglitz et al. 2017; Jagers et al. 2018; Köppl and Schratzenstaller 2023). Plastics have a substantial detrimental impact on the environment, releasing greenhouse gases as they decompose in both terrestrial and marine environments (Royer et al. 2018; Groh et al. 2019). Additionally, marine (micro)plastics negatively affect phytoplankton communities, destabilizing marine ecosystems and contributing to greenhouse gas emissions (Shen et al. 2020). Thus, a tax on plastics could serve as an impactful policy instrument (e.g., Martinho et al. 2017). Finally, given the significant environmental impact of meat compared to plant-based alternatives, research has highlighted the lack of regulatory attention to meat consumption (Poore and Nemecek 2018; Funke et al. 2022; Jagers et al. 2024). Therefore, this research focuses on taxes targeting carbon emissions, plastics, and meat consumption.

Past research has advanced knowledge of the determinants that predict support for environmental policies, such as people’s values, climate change beliefs, and demographic characteristics (e.g., Bergquist et al. 2022; Ejelöv and Nilsson 2020). Another line of research focuses on how policies should be designed to gain support. This line of research has examined several design strategies to encourage support for carbon taxes, such as ear marking funds, implementing trial periods, and sharing information related to the policy (e.g., Carattini et al. 2018; Klenert et al. 2018). More specifically, Carattini et al. (2015) compared support for different versions of a carbon tax, finding the strongest support for taxes that lowered income taxes, redistributed revenues domestically to individual, or earmarked funds for mitigation projects. Recent conjoint experiments have further shown that support for carbon taxes is higher when revenues are recycled for environmental purposes (Malberba et al. 2024), that support for meat tax is greater when justified by animal welfare rather than climate change mitigation (Perino and Schwickert 2022) and that the public is actually not less positive towards fossil fuel subsidy removal compared to carbon taxation (Harring et al. 2023).

In advancing the current state of knowledge about support, experimental research is needed both to establish causality and to study established determinants in isolation (Kallbekken 2023). In this paper, I design a series of experiments comparing the impact of psychological barriers that might limit support for environmental policies. While these policies are tested in an isolated experimental setting, understanding the causal effects of potentially detrimental policy solutions is of practical importance. Properly identifying the most significant barriers to support can guide policymakers and practitioners in avoiding counterproductive strategies. For example, should policymakers focus on making policy proposals as affordable as possible, as effective as possible, or as fair as possible?

Based on meta-analytic evidence and recent research (Bergquist et al. 2022; Ejelöv and Nilsson 2020; Coleman et al. 2023; Keiser et al. 2023), I compare policy proposals that are designed to be costly, ineffective, or unfair to assess the detrimental impact of each barrier on support. This research is guided by two main questions: (1) Are people more reluctant to support unfair policies than costly or ineffective ones? (2) What are the consequences of perceived unfairness?

## Materials and methods: Explorative experiments 1

### Design

In a between-subjects design experiment, participants were randomly presented with one of four policy proposals (control, high price, ineffective, or unfair) of either a plastic tax or a tax on meat. Participants were then asked to:

1. Rate their support for the proposed tax.
2. Modify the proposed policy to increase support.
3. Rate their support for the modified tax.

The aim of letting participants modify the tax and rate their support for the modified tax proposals was to (1) explore participants' ideal distribution of price, ineffectiveness, and fairness (2) explore support for such an ideal proposal. Hence, examine the level of support for a tax where participants had been allowed to remove the barriers associated with cost, ineffectiveness, and unfairness.

### Participants

Two hundred and nineteen participants located in the USA were recruited via Amazon's Mechanical Turk (MTurk). Eleven participants were excluded due to failing the attention check, resulting in a final sample of 208 participants. They were assigned to rate their support for either a tax on plastics or a tax on meat.

### Stimulus material and measures

The taxes were described by specifying the cost of the tax, the tax's effect in decreasing carbon emissions, and how much tax different groups would be pay. Participants in the control condition were informed that the plastic/meat tax would:

1. Be distributing the costs equally (50%–50%) between citizens and corporations, and the poor and the rich.
2. Increase the cost of plastics/meat by 5%.
3. Decrease carbon emissions by 5% by 2030.

For participants in the costly, ineffective, and unfair conditions, however, the tax was described as either increasing costs by 100%, decreasing emissions by 0.000001%, or distributing the costs unequally (75–25% tax distribution: making citizens and the poor pay more than corporations and the rich). Hence, manipulated either cost, effectiveness, or fairness while holding the other predictors constant.

Participants rated their support for the proposed policy using a single item: “Would you support the plastic/meat tax policy?” with response options of 0) “No, I would not support it” and 1) “Yes, I would support it”.

After rating their support for the proposed policy, participants were asked, “Please rate if, and how, you would like to modify the plastics/meat tax policy to make it (more) acceptable for you.” Participants were provided with four sliders to modify cost, effectiveness, and fairness on a scale from 0 to 100. Finally, participants were presented with their own modified version of the proposed policy and asked to rate their support for it. (See examples in Appendix S1).

## Results and discussion: Explorative experiments 1

Compared to the control condition, planned comparisons chi-square analyses revealed substantially lower support for the unfair tax (plastic tax: *χ*^2^(34) = 8.57, *p* = .003, *φ* = .50, meat tax: *χ*^2^(65) = 10.00, *p* = .002, *φ* = .39). However, no statistically significant differences in support were found for either the ineffective (plastic tax: *χ*^2^(42) = 0.32, *p* = .57, *φ* = .08, meat tax: *χ*^2^(69) = 1.79, *p* = .18, *φ* = .16) or the costly (plastic tax: *χ*^2^(39) = 0.74, *p* = .39, *φ* = .14, meat tax: *χ*^2^(68) = 2.61, *p* = .11, *φ* = .20) policy proposals compared to the control condition.

**Fig. 1:**
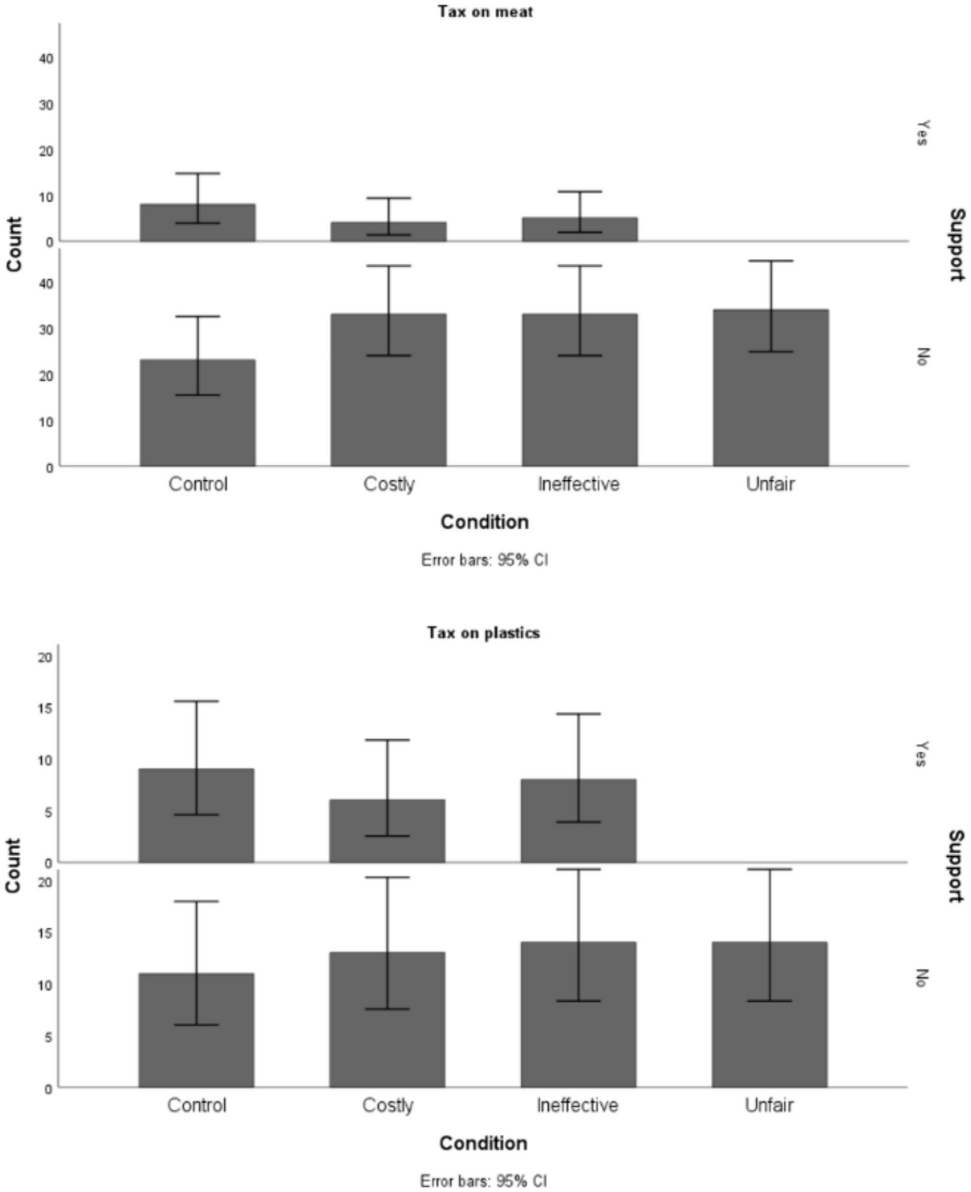
Frequency of participants supporting the proposed environmental taxes across experimental conditions

As depicted in Fig. 1, the lack of support for the unfair policy was striking, with no participants endorsing the unfair tax. These results provide an initial indication that perceived unfairness may be a more potent barrier to supporting environmental policies than cost and ineffectiveness.

In assessing support for the modified policy proposals, McNemar’s tests showed that the modified policies reached stronger support than the proposed policies for both the tax on plastics (*χ*^2^(75) = 19.15, *p* < .001), and for the meat tax (*χ*^2^(140) = 61.97, *p* < .001). Support increased from 31 to 72%, and from 14 to 64%, for the respective taxes. Importantly, levels of support for the modified policy proposals did not differ across conditions in either the plastic tax (*χ*^2^(75) = 6.2, *p* = .10, *φ* = .29) or the meat tax (*χ*^2^(140) = 3.63, *p* = .30, *φ* = .16). These results suggest that support was strengthened when participants modified the policy proposals by removing the barriers of unfairness, extensive costs, and ineffectiveness. It is, however, important to note that even when rating an ideal (and possibly even uncredible policy) a substantial number of participants did not accept it.

Finally, I explored how participants modified the policy proposals (see Fig. S1). The modified policies were set to distribute the tax between the citizens and corporations to M = 31–69% for the plastic tax and M = 28–72% for the meat tax. Similarly, the distribution between the poor and rich was set to 30–70% for the plastic tax and 29–71% for the meat tax. The cost was set at 15% and 8%, and effectiveness was set at 25% and 20% for the plastic tax and meat tax, respectively. Nevertheless, Experiment 1 is limited by not explicitly assessing to what extent participants perceived the modified taxes as credible.

Experiments 1 suggest that perceived unfairness is a stronger barrier to supporting environmental taxes than both costly and ineffective policy proposals. However, these results should be interpreted with caution. The study might have been insufficiently powered to detect the effect of the costly and ineffective policy proposals, and the stimulus material has not been validated. To address the latter of these concerns, a pilot study is conducted to assess the construct validity of the policy proposals.

## Materials and methods: Pilot study

Experiment 1 relied on the manipulations of cost, ineffectiveness, and unfairness to indeed be perceived as costly, ineffective, and unfair, respectively. Manipulations of psychological constructs are, however, rarely safe to assume (Ejelöv and Luke 2020). Therefore, I ran a pilot study to assess both the construct validity and the causal efficacy of the stimulus material.

### Design

In a repeated measures-design, participants were presented with each of four versions of the plastic tax (control vs. costly vs. ineffective vs. unfair) in random order and asked to assess if the policy proposals were: (1) effective in reducing carbon emissions, (2) ineffective in reducing carbon emissions, (3) an acceptable price increase, (4) a too high price increase, (5) fair, and (6) unfair.

### Participants

Powered to detect an effect size *f* = .15 (*α* = .05, 1 − *β* = .95), 88 participants, located in the USA (9 excluded due to failing the attention check) were recruited via MTurk.

### Stimulus material

The pilot study assessed the stimulus material used in Experiment 1. For each policy proposal, participants were asked “*Please rate the proposed tax. According to me, the tax is…*” (1) “*effective in reducing carbon emissions*”, (2) “*ineffective in reducing carbon emissions*”, (3) “*acceptable price increase*”, (4) “*too high price increase*”, (5) “*fair*”, and (6) “*unfair*” on a scale ranging from “*—(4) very strongly disagree”* to “*(4) very strongly agree”*. In addition, to validate the control conditions, participants were asked “*According to you, what would be a fair policy*, …*an effective policy*, and …*what would be an acceptable price for the policy*?” Participants were then presented with the same scales as used in Experiments 1 and 2, ranging from 0 to 100 (now anchored at 0).

## Results and discussion: Pilot study

**Fig. 2:**
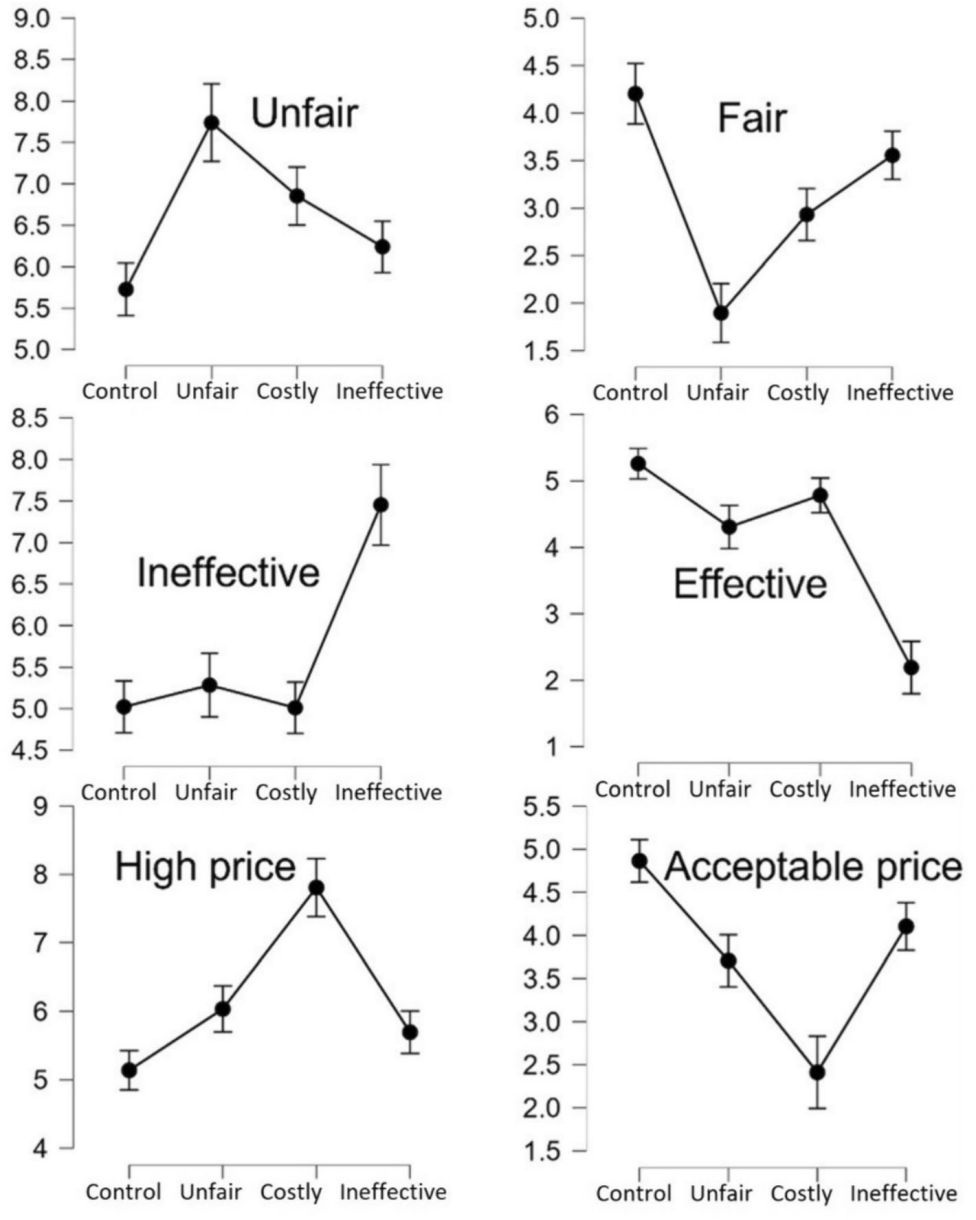
Ratings of (un)fairness, (in)effectiveness, and cost in the four experimental conditions. Error bars represent 95% CI

In assessing how the policy proposals were perceived, I ran a series of repeated measures ANOVAs (all *p’*s < .001, see Fig. 2). Compared to the control condition, ratings of the policy proposals showed the following pattern: The unfair policy proposal was perceived as the most unfair (*d* = 0.87, 95% CI [0.52, 1.22]) and least fair (*d* = 1.11, 95% CI [0.76, 1.46]). The ineffective policy proposal was perceived as the most ineffective (*d* = 1.05, 95% CI [0.68, 1.43]), and least effective (*d* = 1.47, 95% CI [1.06, 1.88]). Finally, the costly policy proposal was perceived as the most expensive (*d* = 1.16, 95% CI [0.79, 1.53]) and was perceived as the proposal with the least acceptable price (*d* = 1.07, 95% CI [0.73, 1.41]). These results corroborate the validity of the stimulus material.

## Materials and methods: Experiment 2

The aim of Experiment 2 was to test the robustness of the findings from Experiment 1, which were limited by a lack of a clear sample size rationale and being exploratory (Lakens 2022). Experiment 2 was a pre-registered and adequately powered study aiming to assess the reliability of the results obtained in Experiment 1. Experiment 2 assessed a tax on plastics and employed the same basic between-subjects design as Experiment 1. The experimental conditions were operationalized as in Experiment 1, while the control condition was modified based on results from the pilot study. As an attention check, participants were asked “Please describe the target of the proposed policy” provided with a text box. Participants failing the attention check were excluded from the final sample.

### Sample size justification

Experiment 2 was powered to detect an effect size of least interest (*w* = 0.20). A power calculation using G*Power (Tails = 2, Effect size *w* = 0.20, *α* = .05, 1 − *β* = .80, *df* = 1) indicated a sample of 197 for each pairwise comparison, resulting in a total sample of 394 participants. Due to the potential attrition from the attention checks, the sample was over-recruited to collect data from 500 participants. According to the pre-registration, if more than 106 participants are excluded in the first data collection, a new sample of 100 participants would be recruited, and so on until reaching at least 394 participants.

### Participants

The final sample consisted of 395 participants recruited from MTurkers, located in the USA. In the sample 55.4% were males. For political preference, assessed via 1) “strongly liberal” to 7) “strongly conservative”, the sample was leaning slightly towards being liberal (M = 3.56, SD = 1.83). See Table S1 for distribution across conditions.

## Results and discussion: Experiment 2

Results from a logistic regression analysis revealed a statistically significant model, (χ^2^(3, N = 395) = 58.45, *p* < .001), indicating that the predictors reliably distinguished between the categories of the outcome variable. The model accounted for 19% of the variance in support (Nagelkerke *R*^2^ = .19).

**Fig. 3:**
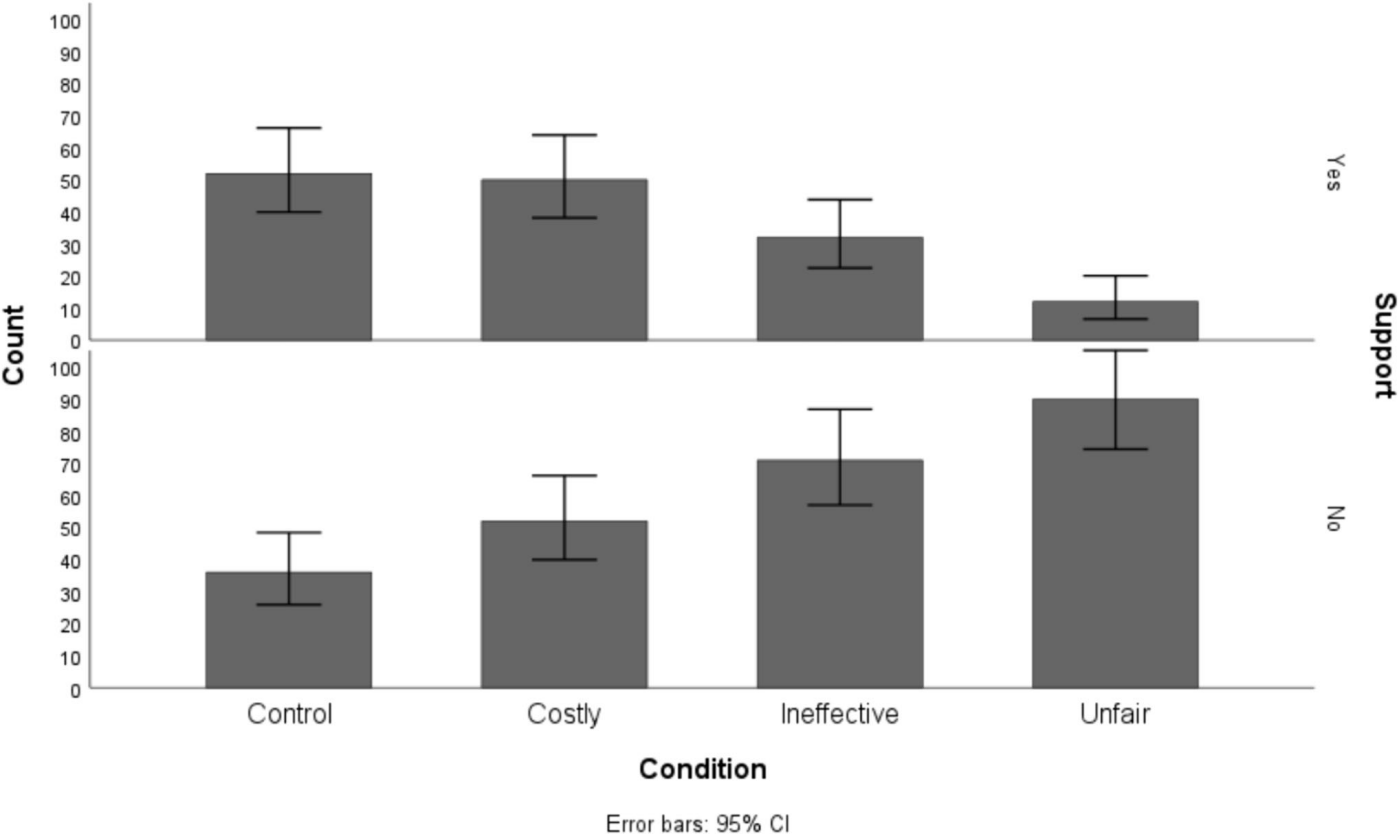
Number of participants (not) supporting the tax on plastics across policy proposals

The model was run using the control condition as the reference (59.1% support). First, when presented with the costly tax, participants levels of support were not statistically significantly lower than the reference (Wald χ^2^ = 1.92, *p* = .16, OR = 1.5, 95% CI [0.85, 2.67]). Descriptively, however, support was reduced by 10 percentage points. When presented with an ineffective tax, support was reduced by 28 percentage points, yielding a statistically significant effect (Wald χ^2^ = 14.69, *p* < .001, OR = 3.21, 95% CI [1.77, 5.81]). Finally, in line with the explorative experiments, unfairness was once again identified as the strongest barrier to support (Wald χ^2^ = 40.13,* p* < .001, OR = 10.83, 95% CI [5.18, 22.64], see Fig. 3). When proposing an unfair tax, participants' levels of support were reduced by 47 percentage points.

**Fig. 4:**
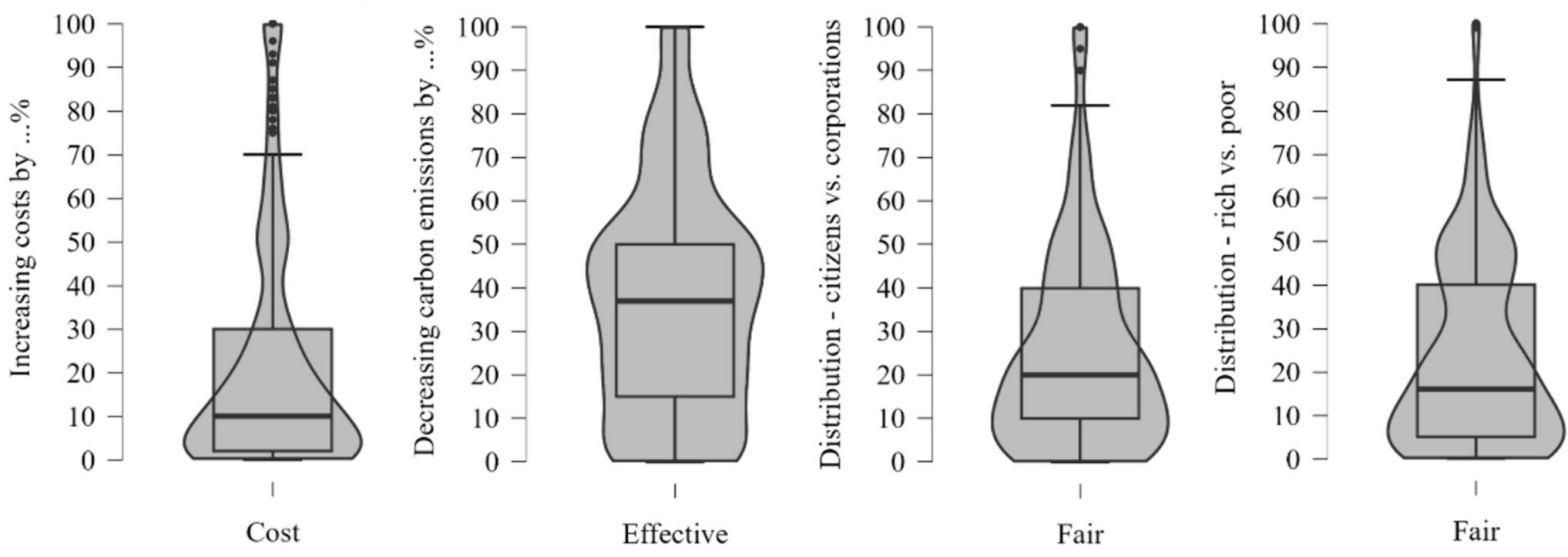
How participants modified the cost, and the effectiveness and distributive fairness of the proposed plastic tax

For the modified tax, results provided no statistically significant results when comparing the baseline condition to the price condition (Wald χ^2^ = .74, *p* = .39), the ineffective condition (Wald χ^2^ = 2.09, *p* = .15), and the unfair condition (Wald χ^2^ = .26, *p* = .61, Fig. 4).

## Materials and methods: Experiment 3

Experiment 2 replicated the effects observed in the exploratory experiment. However, the sample size was determined by a power calculation rather than being representative of the US population. Consequently, the sample was collected from a single platform, Amazon's Mechanical Turk, and may not be representative. In Experiment 3, I aim to replicate these results using a representative sample of the US population, recruited via the platform Prolific Academic (see Table S2). Additionally, the sample size will be increased to over three times that of Experiment 2, with 1400 participants recruited.

Another limitation of Experiments 1 and 2 was their focus on the plastic tax and meat tax. In Experiment 3, I will broaden the scope by assessing a carbon tax. Carbon taxation is widely regarded as one of the most cost-effective methods for reducing greenhouse gas emissions and is frequently recommended by environmental economists and policy researchers (e.g., Sterner and Coria 2012; Stiglitz et al. 2017; Jagers et al. 2018).

A third limitation of Experiments 1 and 2 was a vague formulation when assessing unfairness. More specifically, participants were informed that "*the tax will be allocated to citizens by X% and to poor people by Y%*". This might have led some participants to interpret the tax in terms of revenues, while others might have interpreted it as tax incidence. To clarify, this formulation was revised in Experiment 3, stating that *“The tax will be (1) allocated so that citizens* *pay less* *than corporations (25–75%) and (2) poor* *pay less* *than rich people (25–75%)”*.

### Design

Experiment 3 used the same between-subjects basic design as Experiment 2, while now restricted to measure support for the proposed policy. That is, participants did neither modify the tax nor rate their modified tax. In a between-subjects design, participants were presented with one of four versions of the carbon tax (control vs. costly vs. ineffective vs. unfair) and were then asked to rate their support for a proposed carbon tax, using the same measures as Experiments 1 and 2.

### Participants

The final sample consisted of 1398 participants recruited from Prolific Academic, located in the USA. Two participants, who stated being under 18 years of age were excluded. In the sample, 48% identified as males, 50.9% as women, 1% as non-binary/third gender, and 0.1% preferred not to say. For political preferences, 40.5% of the participants were liberal, 34.8% conservative, and 24.7% independent (answering 4 on the 7-point scale). For more detailed information about the demographics, see Appendices S2–S3.

## Results and discussion: Experiment 3

A logistic regression revealed that the model was statistically significant, (χ^2^(3, N = 1398) = 304.89, *p* < .001), indicating that the predictors reliably distinguished between the categories of the outcome variable. The model accounted for 26% of the variance in support (Nagelkerke *R*^2^ = .26).

**Fig. 5:**
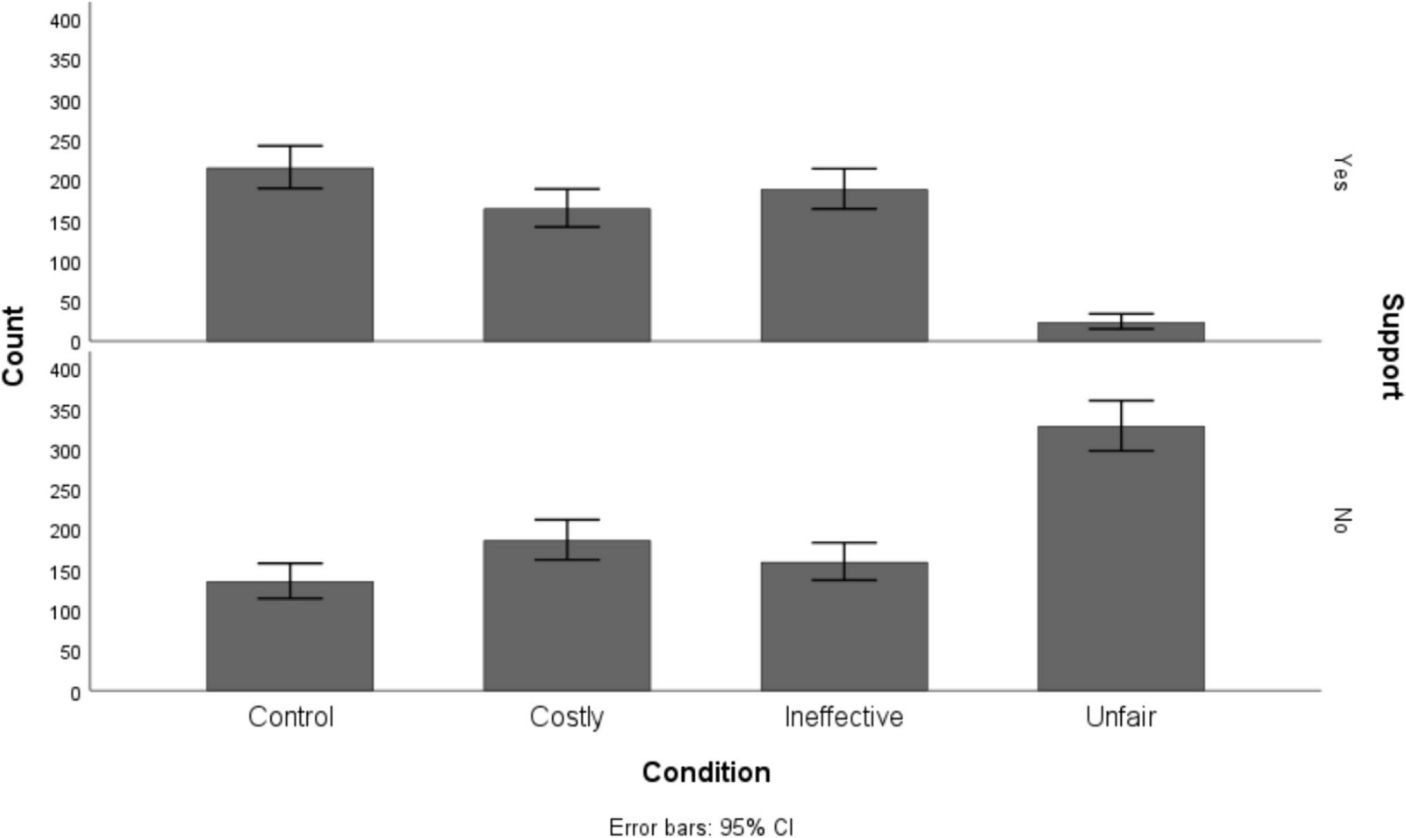
Number of participants (not) supporting the carbon tax across policy proposals

The model used the control condition as the reference (61.4% support). When presented with a costly carbon tax, participants support was reduced by 15.5 percentage points (Wald χ^2^ = 14.86, *p* < .001, OR = 1.8, 95% CI [1.43, 2.44]). In the ineffective condition, support was reduced by 7.2 percentage points (Wald χ^2^ = 3.75, *p* = .053, OR = 1.35, 95% CI [0.99, 1.82]). Once again, the lack of support for an unfair carbon tax was apparent (Wald χ^2^ = 166.46,* p* < .001, OR = 22.71, 95% CI [14.13, 36.50]. When proposing an unfair carbon tax, support was reduced by 54.8 percentage points compared to the reference condition. Describing these data frequencies, 328 of 351 participants would not support an unfair carbon tax (see Fig. 5).

One limitation of Experiments 1–3 is that fairness was operationalized solely as distributional fairness, while fairness is indeed a multifaceted concept including both distributional and procedural aspects (e.g., Heyen 2022). In Experiment 4, I will assess these dimensions of (un)fairness and explore if an unfair policy proposal affects perceived ineffectiveness and trust. Another limitation is that Experiments 1–3 were based on samples from the United States. Cultural experiences may shape how unfairness is perceived (e.g., Maresto and Zeidler 2022). Therefore, Experiment 4 will recruit participants from three different cultures: the USA, the UK, and India.

## Materials and methods: Experiment 4

Experiments 1–3 consistently showed that unfairness was the strongest barrier to supporting environmental taxes. However, fairness is a multifaceted concept including both procedural and distributional aspects (Maestre-Andrés et al. 2019).

Experiment 4 was pre-registered and adequately powered to test the effects of procedural and distributional fairness on support for a carbon tax across three cultures (the USA, the UK, and India). Furthermore, survey data suggest that perceived unfairness is related to both trust and effectiveness (Povitkina et al. 2021; Bergquist et al. 2022). Therefore, I will explore if experimentally induced unfairness affects people’s ratings of trust in policymakers and the perceived effectiveness of the policy proposal.

### Design

Experiment 4 used a 2 (distributional: fair vs. unfair) × 2 (procedural: fair vs. unfair) between-subjects design, assessing support for a proposed carbon tax, measured on the same scale as Experiment 1–3 (See also Appendix S4).

### Explorative analyses

In addition to the detrimental effects on support, I will explore if unfair policy proposals also affect participants’ trust in policymakers and the perceived effectiveness of the policy. Both trust and effectiveness have shown to be key determinants for support (e.g., Kallbekken et al. 2013; Bergquist et al. 2022). In assessing trust and effectiveness, participants were once again shown the policy proposal, and asked “*do you*…”, followed by six items, measuring trust (using 1 item: “*trust the policy-makers who will implement the tax?”*), effectiveness (using 2 items: “*believe that the tax is effective in reducing carbon emissions?”* and *“believe that the tax will reduce carbon dependent behavior?”*). Furthermore, as a manipulation check of perceived unfairness, I assessed procedural fairness (using 1 item: *“believe that the tax is fairly implemented?”*), and distributional fairness (using 2 items: *“believe that the tax is distributed fairly between the rich and the poor?”,* and *“believe that the tax is distributed fairly between corporations and citizens?”*). Participants answered on a 7-point scale ranging from − 3 *strongly disagree* to 3 *strongly agree*.

### Sample size justification

In Experiment 4, the effect size for the difference in support of the proposed policy between the control condition and unfair condition was *w* = .50. The effect might, however, be weaker for procedural unfairness, I will therefore power to detect an effect size of *w* = .40, with an alpha level corrected for multiple comparisons (one for each country). A power calculation using G*Power (Tails = 2, Effect size *w* = 0.40, *α* = 0.0125, 1 − *β* = .80, *df* = 1) indicated a sample of 70 for each pairwise comparison, resulting in a total sample of 140 participants for each country. Due to attrition from the attention checks, I will over-recruit and collect data from 200 participants. Participants in experiments 1–3 were located in the USA. To improve the external validity of these results, Experiment 4 will include participants located in the USA, the UK, and India. Implying a total sample of at least 420 participants.

### Participants

The final sample was 495 participants recruited via MTurk, located in the USA (n = 197), UK (n = 152), and India (n = 146). Due to failed attention checks, 3 of 200 participants were excluded from the US-American sample, 34 of 186 participants from the UK sample, and 94 of 250 from the Indian sample. For more detailed information about the demographics, see Table S2.

## Results and discussion: Experiment 4

First, in validating the stimulus material I ran a 2 (distribution: fair vs. unfair) × 2 (procedural: fair vs. unfair) MANOVA assessing the three manipulation checks of perceived unfairness. In assessing procedural unfairness, results showed a main effect of procedural unfairness (*F*(1, 491) = 5.94, *p* = .015, *η*_*p*_^2^ = .01), indicating that procedurally unfair was successfully manipulated. Importantly, results also showed a main effect of distributional unfairness *F*(1, 491) = 132.17, *p* < .001, *η*_*p*_^2^ = .21), but no statistically significant interaction (*p* > .05). The two items measuring perceived distributional unfairness showed acceptable reliability and was thus transformed to an index variable (Cronbach’s alpha = .93). In assessing distributional unfairness, results showed a main effect of distributional unfairness (*F*(1, 491) = 172.08, *p* < .001, *η*_*p*_^2^ = .26), indicating that distributional unfair was successfully manipulated. Results showed no significant main effect of procedural unfairness and no significant interaction (*p* > .05). Taken together, these data support the construct validity of the induced distributional and procedural unfairness.

Results from a logistic regression analysis revealed that the model was statistically significant, (χ^2^(3, N = 495) = 157.69, *p* < .001), indicating that the predictors reliably distinguished between the categories of the outcome variable. The model accounted for 36% of the variance in support (Nagelkerke *R*^2^ = .36).

The model was run using the fair distribution and fair procedure (i.e. fair condition) as the reference condition. Support decreased when presenting a carbon tax with an unfair distribution and fair procedure (Wald χ^2^ = 70.04, *p* < .001, OR = 0.08, 95% CI [0.04, 0.14]). Descriptively, support was reduced by 56.5 percentage points compared to the fair condition. These effects were consistent across countries the three countries USA (Wald χ^2^ = 34.95, *p* < .001, OR = 0.03, 95% CI [0.01, 0.10]), UK (Wald χ^2^ = 21.97, *p* < .001, OR = 0.07, 95% CI [0.02, 0.22]), and India (Wald χ^2^ = 11.52, *p* < .001, OR = 0.17, 95% CI [0.06, 0.47]).

In the procedural unfair condition, however, support was reduced by 8.6 percentage points, yielding a non-statistically significant effect (Wald χ^2^ = 2.46, *p* = .12, OR = 0.63, 95% CI [0.35, 1.12]). These effects were also consistent across countries the three countries USA (Wald χ^2^ = 3.71, *p* = .054, OR = 0.42, 95% CI [0.17, 1.02]), UK (Wald χ^2^ = 0.44, *p* = .51, OR = 0.70, 95% CI [0.25, 1.01]), and India (Wald χ^2^ = 0.33, *p* = .57, OR = 1.46, 95% CI [0.40, 5.35]).

Finally, support was reduced by 60.1 percentage points when in the distributionally unfair and procedurally unfair conditions. Yielding a statistically significant effect (Wald χ^2^ = 78.98, *p* < .001, OR = 0.06, 95% CI [0.03, 0.11]), which was robust across the three countries USA (Wald χ^2^ = 14.38, *p* < .001, OR = 0.01, 95% CI [0.001, 0.05]), UK (Wald χ^2^ = 26.16, *p* < .001, OR = 0.03, 95% CI [0.01, 0.12]), and India (Wald χ^2^ = 7.66, *p* = .006, OR = 0.24, 95% CI [0.09, 0.66]).

Taken together, these results show that distributional unfairness was a stronger psychological barrier for supporting a carbon tax than procedural unfairness. This pattern was consistent for participants for the USA, the UK and India.

Finally, do unfair policy proposals affect trust in policymakers and the perceived effectiveness of the policy? Results from a 2 (distribution: fair vs. unfair) × 2 (procedural: fair vs. unfair) MANOVA showed that trust in policymakers was lower for participants receiving a distributionally unfair policy proposal (*F*(1, 491) = 74.69, *p* < .001, *η*_*p*_^2^ = .13) and for participants who received a procedurally unfair policy proposal (*F*(1, 491) = 5.89, *p* = .016, *η*_*p*_^2^ = .01). It should be noted that the effect size of distributional unfairness is substantially larger than the effect size of procedural unfairness. This once again suggests that distributional unfairness, as operationalized in the current experiment, was more detrimental than procedural unfairness. The unfair policy proposal also affected participants perceived effectiveness of the carbon tax. After receiving a distributionally unfair policy proposal, participants rated the policy as ineffective both in reducing CO_2_ emissions (*F*(1, 491) = 66.48, *p* < .001, *η*_*p*_^2^ = .12), and reducing carbon-dependent behaviors (*F*(1, 491) = 86.16, *p* < .001, *η*_*p*_^2^ = .15). No statistically significant main effect of procedural unfairness (*p* > .05) or interaction (*p* > .05) was found. Taken together, these data showed that participants who received an unfair carbon tax proposal both perceived the policy as less effective and reported lower trust in policymakers than those who received a fair carbon tax proposal. In terms of psychological processes, the effect could be a result of post-decisional cognitive dissonance, where participants' rating of (non)support motivated them to rate the related attitudes towards policymakers and effectiveness consistently (e.g., Enisman et al. 2021). However, cognitive dissonance doesn’t sufficiently explain why the effects were substantially larger for distributional unfairness compared to procedural unfairness.

## General Discussion

In a series of experiments, I report that perceiving an environmental tax as unfair is a stronger barrier to support than perceiving it as costly or ineffective. After conceptually refining unfairness, distributional unfairness was found to be a stronger barrier than procedural unfairness. Finally, the detrimental role of unfairness not only severely limited support but also made participants express lower trust in policymakers and made them perceive a carbon tax as less effective in reducing CO_2_ emissions and promoting sustainable behaviors. The detrimental effect of perceived unfairness for environmental policy support was consistently found across four experiments (1) assessing taxes on meat, plastics, and carbon, (2) using a large US-representative sample, and (3) recruiting participants from USA, UK, and India.

These results advance past research, emphasizing the role of (un)fairness in supporting environmental policies (e.g., Bergquist et al. 2022), by demonstrating that experimentally induced unfairness is a substantial barrier to supporting such policies. This is important as past research on determinants for accepting environmental policies is largely correlational, implying that the potentially directional effect of fairness is unclear. More specifically, it is unclear if a correlation between fairness and support implies that “fairer” policies will increase support, or if people will rationalize their stated (un)support by aligning it with perceived (un)fairness (e.g., Festinger 1957; Regan and Kilduff 1988; Jarcho et al. 2011).

This research provides experimental evidence that induced unfairness is a stronger barrier for supporting environmental taxes than induced costs and ineffectiveness. First, these experiments suggest that unfairness is a substantial barrier to support, whereas reestablished fairness to some extent promotes support. Second, Experiment 4 suggests that distributional unfairness is a stronger barrier than procedural unfairness. Additionally, an unfair policy proposal reduced participants’ trust in policymakers and made them perceive the policy as ineffective. It should however be noted that cost and effectiveness also reduced support statistically significantly in Experiment 3, which was based on a representative sample and had the highest levels of statistical power. With that said, results from experiments 1 and 2 are descriptively consistent, also showing detrimental effects of cost and ineffectiveness, as observed in past research (e.g., Keiser et al. 2023). In terms of effect sizes, however, the role of unfairness was consistently found to be more important than both cost and ineffectiveness, which is largely in line with meta-analytic findings (e.g., Bergquist et al. 2022).

One limitation of the present research is that distributional unfairness was operationalized to induce extensive costs for some groups (e.g., the poor and citizens). Conceptually, this implies that individual fairness (fair to me) and collective fairness (fair to others) were not separated (Maestre-Andrés et al. 2019). Despite this, induced unfairness resulted in lower support than the costly policy proposal, suggesting that the detrimental effect of supporting an unfairly distributed tax cannot be sufficiently explained by increased costs alone.

Another limitation is the operationalization of procedural fairness. While distributional fairness was measured by concrete distributions of costs between the rich and the poor, and between citizens and corporations, procedural fairness was assessed by asking participants if they “believe that the tax is fairly implemented?” This limitation is partially due to the nature of these constructs. Specifically, when assessing procedures, people might consider whether the process was democratic, ethical, free from biases, trustworthy (in terms of government involvement), etc. In contrast, distributional (un)fairness is fundamentally about the distribution of resources between different groups. Although distributional (un)fairness applies to various forms of resources and groups, it is inherently about people’s perceptions of these distributions. Given the importance of procedural fairness (e.g., Nagin and Telep 2017), the results from Experiment 4 were surprising. Relatedly, although results were consistent across experiments, the current research is limited to using single operationalizations of the assessed constructs. Hence, it is unclear to what extent these results can be generalized to other measures of costs, ineffectiveness, and unfairness. I, therefore, encourage future research to experimentally assess the role of unfairness in different types of procedures associated with environmental policies.

A third limitation is that the operationalization of unfairness could be criticized for favoring a liberal type of unfairness (Graham et al. 2009), as perceived unfairness might differ between conservatives and liberals. I explored this potential limitation by reanalyzing the data. Results showed that participants who rated a 50–50 distribution as fair were more conservative (M = 5.41, SD = 1.54, n = 17) compared to those who rated that the poor should pay less than the rich (M = 3.21, SD = 1.71, n = 66, t(81) = 4.8, *p* < .001, d = 1.31, 95% CI [0.73, 1.87]). This suggests that distributional unfairness was operationalized to resonate with a “liberal interpretation” of unfairness.

Fourth, the modified tax proposals increase support in all conditions. This, by itself, should come as no surprise, as participants tailored the tax to fit their preferences. It is however unclear if, and to what extent such an increase in support was caused by the content of the tax or the fact that participants modified the tax themselves. According to consistency theories, the latter explanation should also be responsible for attitude change (e.g., Festinger 1957; Regan and Kilduff 1988; Jarcho et al. 2011).

A fifth limitation concerns discriminant validity. More specifically, manipulating costs, ineffectiveness, and unfairness, respectively, did not leave other aspects of the policy proposal unaffected (See Fig. 2). For example, an unfair policy seemed to be perceived as more costly than an ineffective policy and a costly policy seemed to be perceived as more unfair than an ineffective policy. Although this does imply that the experimental manipulations were not fully isolated, it also provides valuable insight into the interactions between these aspects of policy proposals. Such interactions lie outside the scope of the present research, yet I strongly encourage future research to examine this in more detail.

Relatedly, I assessed the main effects of cost, ineffectiveness, and unfairness, while in applied settings, these barriers might interact. For example, how important is perceived unfairness for “low-price” taxes compared to “high-price” taxes, or when a policy is perceived as effective compared to ineffective? I encourage future research to experimentally assess the potential interactions among these barriers to support for environmental policies.

Finally, the current experiments assessed support for taxes. It is unclear if these results will hold when assessing environmental laws, and across different types of (more specific) taxes such as land use taxes, pesticide taxes, and congestion charges. There is, however, meta-analytic evidence to support that fairness is a descriptively stronger determinant of support than effectiveness for both regulations and economic policies (Bergquist et al. 2022).

### Practical implications

Throughout four experiments, I aimed to assess the extent to which extensive costs, perceived ineffectiveness, and perceived unfairness are reducing support for various environmental taxes. It should be noted that these studies assessed support using experimental designs, which strengthens control over extraneous factors while weakening ecological validity. When interpreting the current results in the light of reviews and meta-analysis (e.g., Bergquist et al. 2022; Ejelöv and Nilsson 2020), the field provide practical guidelines for policymakers. If faced with a tradeoff between an expensive policy and an unfairly distributed policy, policymakers should probably prioritize avoiding taxes that risk being perceived as unfair.

## Conclusion

Consistent evidence from four experiments suggests that perceived unfairness is a stronger barrier to supporting environmental taxes than both costs and perceived ineffectiveness. When environmental taxes are perceived as unfair, support decrease substantially. The lack of support was more apparent for unfair policies compared to those involving substantial costs or were ineffective in mitigating climate change. Importantly, when modified to reestablish fair distribution, reasonable costs, and effectiveness, environmental taxes garnered support from most participants. In assessing the consequences of unfair environmental policies, I found that participants perceived unfair policies as less effective than fair policies. Finally, in addition to being the strongest barrier to support, proposing unfair policies decreased participants' trust in policymakers.

## Supplementary Information

Below is the link to the electronic supplementary material.

Supplementary file1 (PDF 736 KB)

## Data Availability

Data and materials from all experiments are publicly available at the Open Science Framework: https://osf.io/n9j7q/.
