# Supplementary material for: Limiting support for environmental policies: Unfairness is a more critical barrier than cost and ineffectiveness
Source: Ambio. 2024 Sep 17;54(2):350–63. doi: 10.1007/s13280-024-02074-9 (PMC11662122; doi:10.1007/s13280-024-02074-9)
Supplement: Supplementary file 1 — Supplementary file1 (PDF 736 KB) [file 13280_2024_2074_MOESM1_ESM.pdf]

AMBIO

Supplementary Information

Title: Limiting support for environmental policies: Unfairness is a more critical barrier than cost and ineffectiveness.

Author: Magnus Bergquist

Appendix S1

Example of a proposed policy: Neutral condition

> Please rate the following policy proposal:
>
> A tax on plastics
>
> The tax will be
> 1) allocated equally between citizens and corporations (50-50%)
> and
> 2) equal across poor and rich people (50-50%)
>
> The policy will
> 3) increasing the price of plastics by +5%
> 4) decrease carbon emissions by 5% to 2030.

Example of a modified policy:

When asked "Please rate if, and how, you would like to modify the plastics tax policy to make it (more) acceptable for you." Followed by four specific rating on a scale from 0 to 100.

1) Allocation between: **<u>citizens and corporations</u>** (50 = equal contribution 50%-50%).

   40 = citizens will be taxed **less** than corporations at a rate of 40%-60%

   60 = citizens will be taxed **more** than corporations at a rate of 60%-40%

2) Allocation between: **<u>poor and rich people</u>** (50 = equal contribution 50%-50%).

   40 = citizens will be taxed **less** than corporations at a rate of 40%-60%

   60 = citizens will be taxed **more** than corporations at a rate of 60%-40%

3) **How much higher** should **prices** on plastics be? 0 = 0 % no change , 100 = 100% higher than present

4) **How much** should the tax decrease carbon emission to 2030? 0 = 0% decrease, 100 = 100% decrease

Please rate your modified policy proposal:

A tax on plastics

The tax will be
1) allocated to citizens by 22% and
2) to poor people by 20%

The policy will
3) increasing the price of plastics by 0%
4) decrease carbon emissions by 46% to 2030.

Figure S1. Modification of the proposed policies in Experiment 1

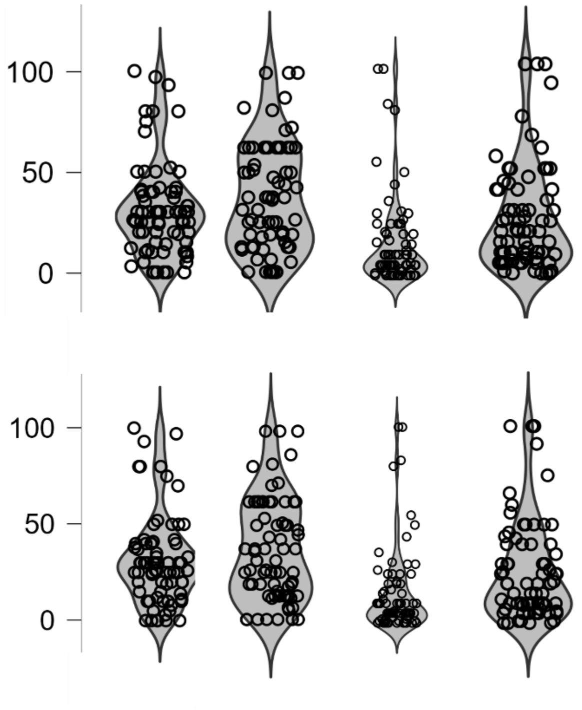

How participants modified the two fairness measures, cost, and the effectiveness of the proposed plastic tax (above) and the meat tax (below).

Table S1: Distribution of political preference and gender across conditions in Experiment 2

Descriptive Statistics

	Political preference			
	Control	Cost	Ineffectiveness	Unfairness
Valid	88	102	103	102
Mean	3.64	3.75	3.52	3.33
Std. Deviation	2.09	1.85	1.73	1.68

The distribution of gender in the conditions was: 61.4% males in control, 52.9% males in cost, 59.2% males in ineffectiveness, and 49% males in unfairness.

Table S2: Specifications of the US representative sample as distributed by Prolific Academic

Sample breakdown

**Date Of Birth**	
18-27	**210**
28-37	**210**
38-47	**196**
48-57	**206**
58-150	**378**
**U.S. Political Affiliation**	
Democrat	**380**
Independent	**510**
Republican	**310**
**Sex**	
Female	**600**
Male	**600**

Appendix S2: Demographics from Experiment 3

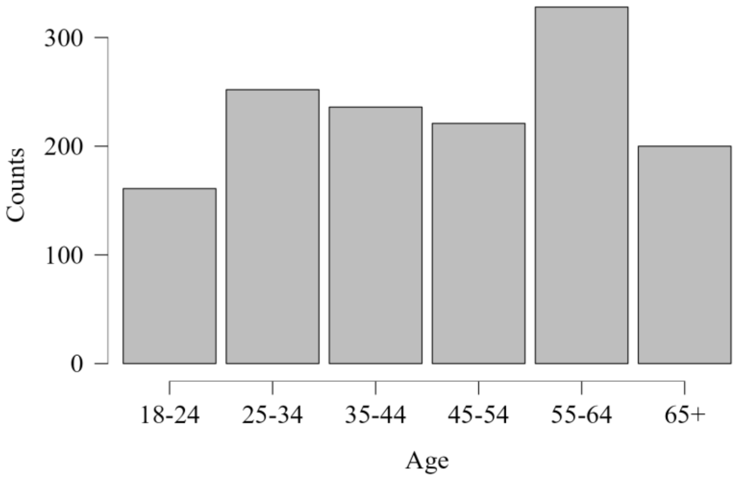

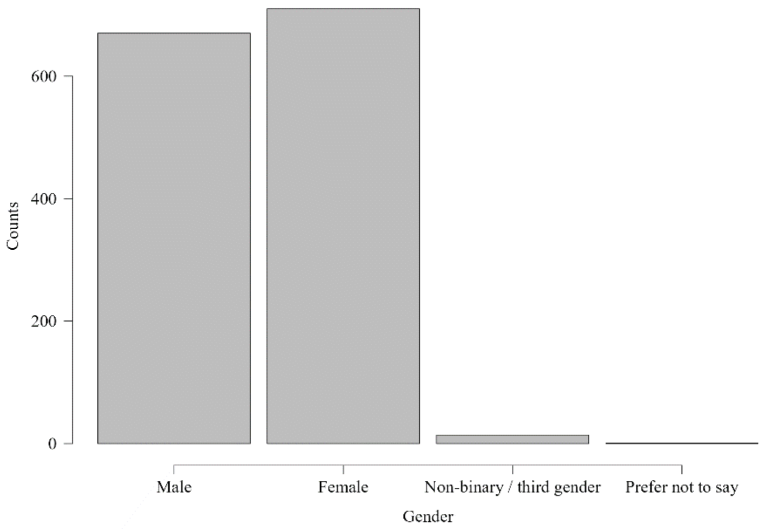
600
400
200
0
Counts
Male
Female
Non-binary / third gender
Prefer not to say
Gender

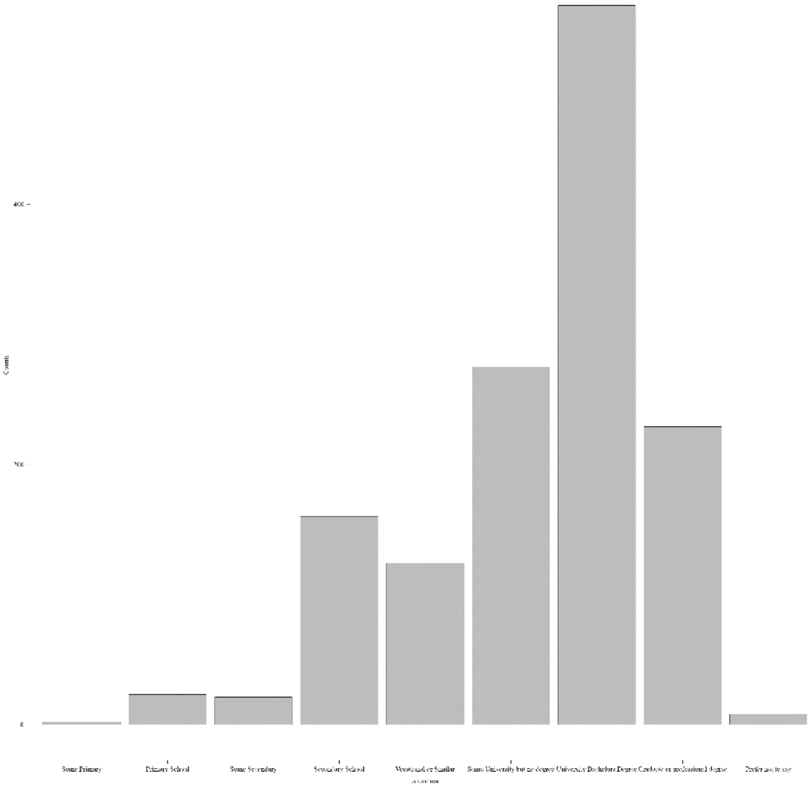
Some Primary
Primary School
Some Secondary
Secondary School
Vocational or Similar
Some University but no degree
University Bachelors Degree
Graduate or professional degree
Prefer not to say

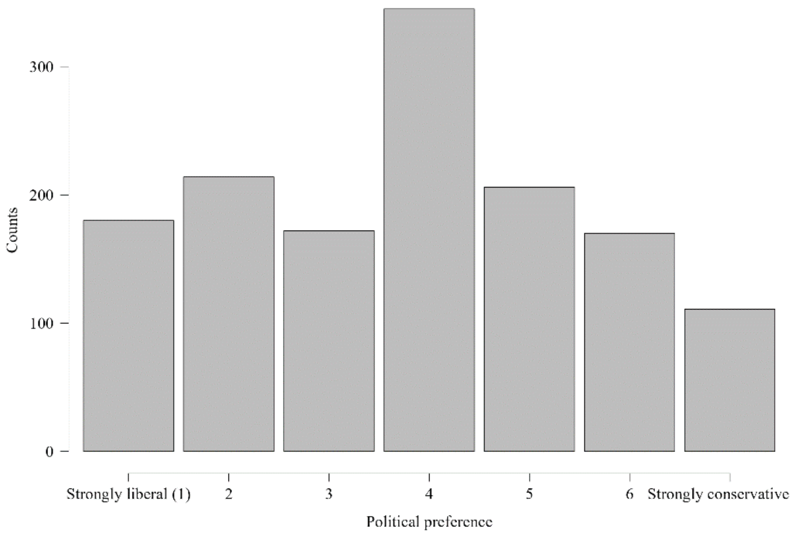
300
200
100
0
Counts
Strongly liberal (1)
2
3
4
5
6
Strongly conservative
Political preference

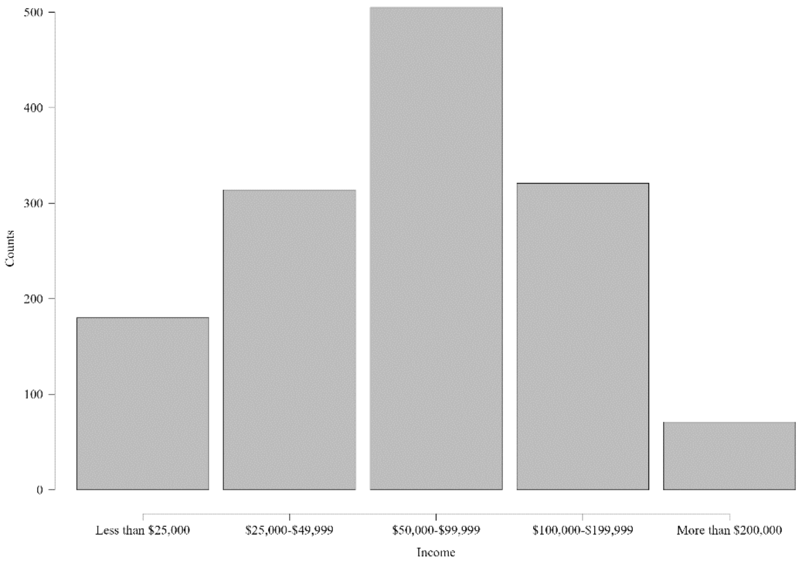
500
400
300
200
100
0
Counts
Less than $25,000
$25,000-$49,999
$50,000-$99,999
$100,000-$199,999
More than $200,000
Income

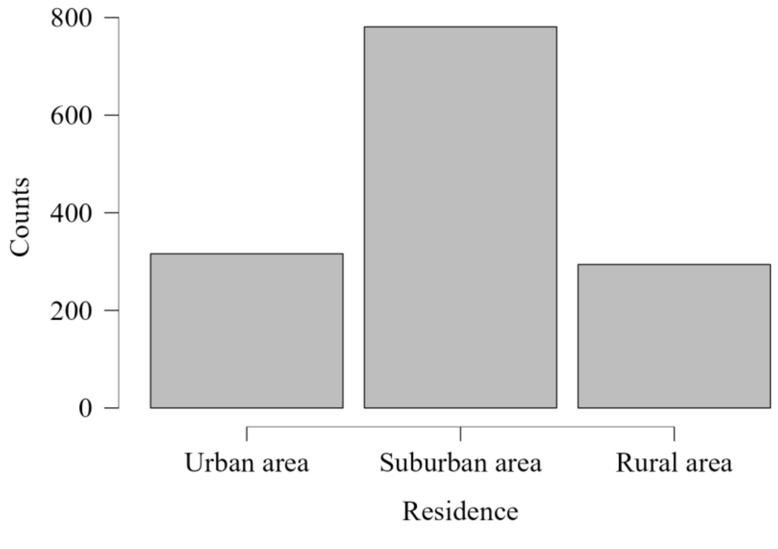
800
600
400
200
0
Counts
Urban area
Suburban area
Rural area
Residence

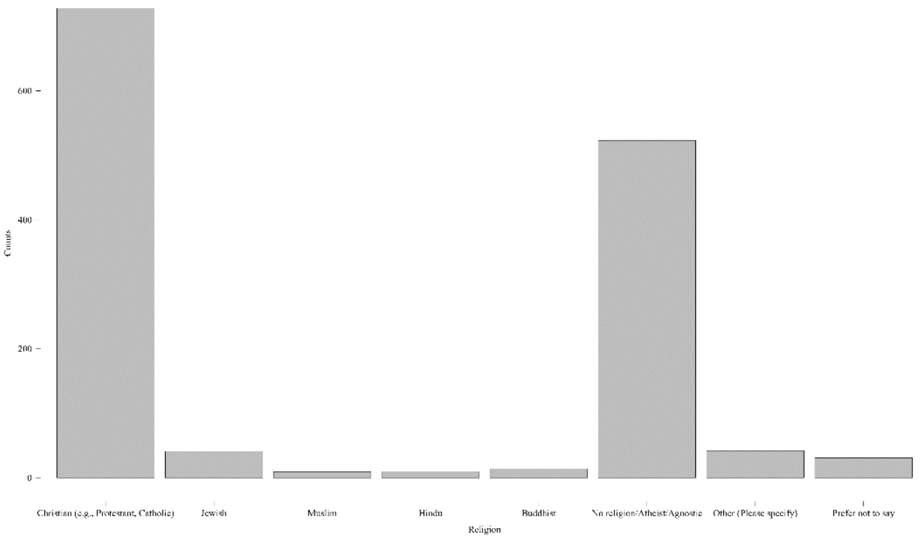
Counts
600
400
200
0
Christian (e.g., Protestant, Catholic)
Jewish
Muslim
Hindu
Buddhist
No religion/Atheist/Agnostic
Other (Please specify)
Prefer not to say
Religion

Appendix S3: Distribution of political preference, age, and gender across conditions in Experiment 3

Descriptive Statistics

	Political preference			
	Control	Cost	Ineffectiveness	Unfairness
Valid	350	350	347	351
Mean	3.81	3.84	3.76	3.84
Std. Deviation	1.77	1.84	1.79	1.78

Control

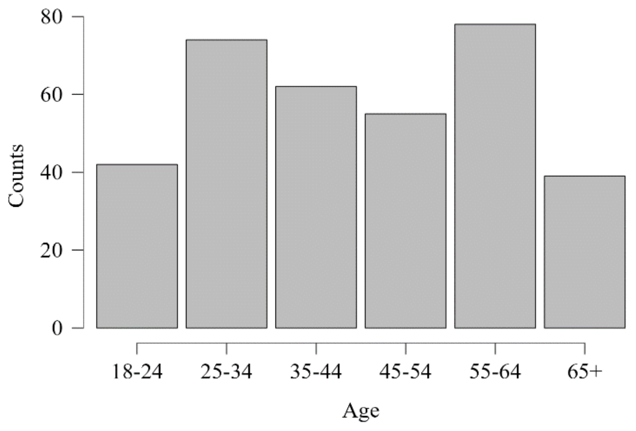

Cost

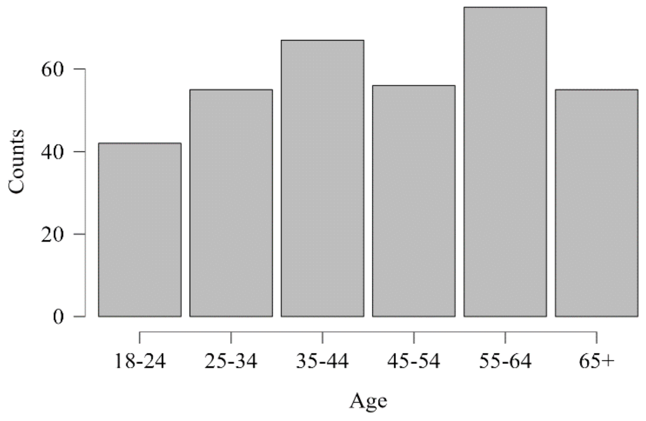

Ineffectiveness

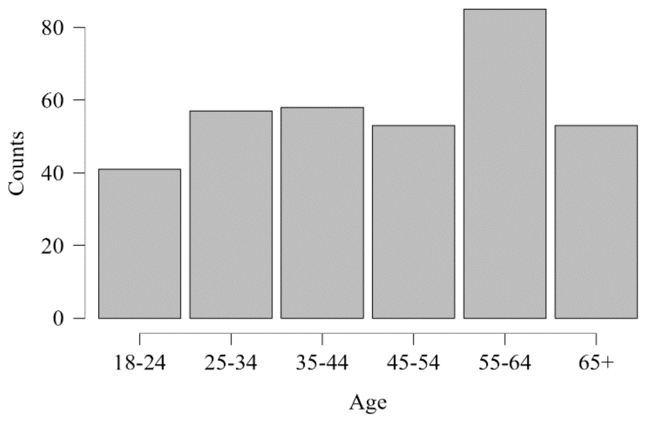

Unfairness

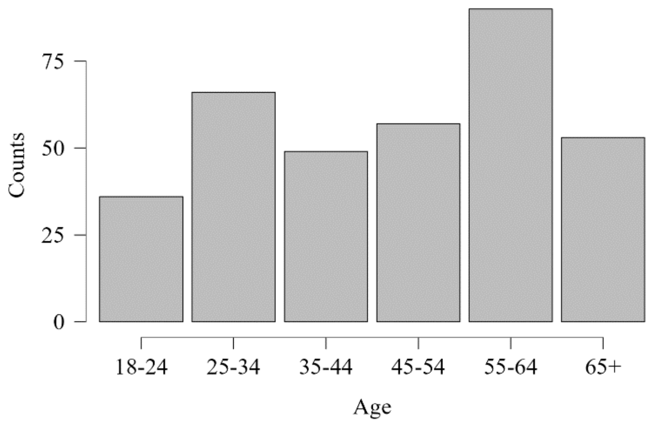

The distribution of gender for each condition was: control (47.7% males, 2.3% non-binary / third gender / prefer not to say) cost (48.7% males, 0.6% non-binary / third gender / prefer not to say) ineffectiveness (49% males, 0.6% non-binary / third gender / prefer not to say), and unfairness cost (46.7% males, 0.9% non-binary / third gender / prefer not to say).

Appendix S4: Stimulus material Experiment 4

Stimulus material from the distributional fair and procedural fair condition:

"*A carbon tax*

The tax will be
1) allocated so that citizens pay ***less*** *than corporations (25-75%) and*
2) poor pay ***less*** *than rich people (25-75%)*

When deciding to implement the policy or not
3) ***all*** *citizens will be able to take part in a decisive referendum (100%)"*

Stimulus material from the distributional unfair and procedural unfair condition:

"A carbon tax

The tax will be
1) allocated so that citizens pay ***more*** *than corporations (75-25%) and*
2) poor pay ***more*** *than rich people (75-25%)*

When deciding to implement the policy or not
3) ***few*** *citizens will be able to take part in a decisive referendum (25%)"*

Table S2: Distribution of political preference and gender across conditions in Experiment 4

Descriptive Statistics

	Political preference			
	Fair Dist + Fair Proc	Unfair Dist + Fair Proc	Fair Dist + Unfair Proc	Unfair Dist + Unfair Proc
Valid	132	122	117	124
Mean	3.80	3.40	3.50	3.76
Std. Deviation	1.73	1.66	1.66	1.91

The distribution of gender in the conditions was: 68.2% males in Fair Dist + Fair Proc, 61.5% males in Unfair Dist + Fair Proc, 63.2% males in Fair Dist + Unfair Proc, and 62.9% males in Unfair Dist + Unfair Proc.

Appendix S5: Deviations from pre-reg

The pre-registrations are available at: https://osf.io/n9j7q/
Data collection in Experiment 4: I could not recruit a sufficient number of participants from Brazil so it was dropped from the data collection. Ending up with three countries: USA, UK, and India. For the Indian sample, I required 50 extra at time two instead of 100, this was because I chose to recruit masters' workers, which conventionally demand higher payment.
